# Time to Endoscopy or Colonoscopy Among Adults Younger Than 50 Years With Iron-Deficiency Anemia and/or Hematochezia in the VHA

**DOI:** 10.1001/jamanetworkopen.2023.41516

**Published:** 2023-11-06

**Authors:** Joshua Demb, Lin Liu, Caitlin C. Murphy, Chyke A. Doubeni, Maria Elena Martinez, Samir Gupta

**Affiliations:** 1 Division of Gastroenterology, Department of Internal Medicine, University of California, San Diego, La Jolla; 2 Jennifer Moreno Veteran Affairs San Diego Healthcare System, San Diego, California; 3 Moores Cancer Center, University of California, San Diego, La Jolla; 4 Herbert Wertheim School of Public Health and Human Longevity Science, University of California, San Diego, La Jolla; 5 University of Texas Health Science Center at Houston (UTHealth Houston) School of Public Health, Houston; 6 Department of Family and Community Medicine of the College of Medicine. Comprehensive Cancer Center, The Ohio State University Wexner Medical Center, Columbus

## Abstract

**Importance:**

To date, the diagnostic test completion rate and the time to diagnostic endoscopy or colonoscopy among adults with iron-deficiency anemia (IDA) and/or hematochezia have not been well characterized.

**Objective:**

To evaluate the diagnostic test completion rate and the time to diagnostic testing among veterans younger than 50 years with IDA and/or hematochezia.

**Design, Setting, and Participants:**

This cohort study was conducted within the Veterans Health Administration between October 1, 1999, and December 31, 2019, among US veterans aged 18 to 49 years from 2 separate cohorts: those with a diagnosis of IDA (n = 59 169) and those with a diagnosis of hematochezia (n = 189 185). Statistical analysis was conducted from August 2021 to August 2023.

**Exposures:**

Diagnostic testing factors included age, sex, race and ethnicity, Veterans Health Administration geographic region, and hemoglobin test value (IDA cohort only).

**Main Outcomes and Measures:**

Primary outcomes of diagnostic testing were (1) bidirectional endoscopy after diagnosis of IDA and (2) colonoscopy or sigmoidoscopy after diagnosis of hematochezia. The association between diagnostic testing factors and diagnostic test completion was examined using Poisson models.

**Results:**

There were 59 169 veterans with a diagnosis of IDA (mean [SD] age, 40.7 [7.1] years; 30 502 men [51.6%]), 189 185 veterans with a diagnosis of hematochezia (mean [SD] age, 39.4 [7.6] years; 163 690 men [86.5%]), and 2287 veterans with IDA and hematochezia (mean [SD] age, 41.6 [6.9] years; 1856 men [81.2%]). The cumulative 2-year diagnostic workup completion rate was 22% (95% CI, 22%-22%) among veterans with IDA and 40% (95% CI, 40%-40%) among veterans with hematochezia. Veterans with IDA were mostly aged 40 to 49 years (37 719 [63.7%]) and disproportionately Black (24 480 [41.4%]). Women with IDA (rate ratio [RR], 0.42; 95% CI, 0.40-0.43) had a lower likelihood of diagnostic test completion compared with men with IDA. Black (RR, 0.65; 95% CI, 0.62-0.68) and Hispanic (RR, 0.88; 95% CI, 0.82-0.94) veterans with IDA were less likely to receive diagnostic testing compared with White veterans with IDA. Veterans with hematochezia were mostly White (105 341 [55.7%]). Among veterans with hematochezia, those aged 30 to 49 years were more likely to receive diagnostic testing than adults younger than 30 years of age (age 30-39 years: RR, 1.15; 95% CI, 1.12-1.18; age 40-49 years: RR, 1.36; 95% CI, 1.33-1.40). Hispanic veterans with hematochezia were less likely to receive diagnostic testing compared with White veterans with hematochezia (RR, 0.96; 95% CI, 0.93-0.98).

**Conclusions and Relevance:**

In the cohorts of veterans younger than 50 years with IDA and/or hematochezia, the diagnostic test completion rate was low. Follow-up was less likely among female, Black, and Hispanic veterans with IDA and Hispanic veterans with hematochezia. Optimizing timely follow-up across social and demographic groups may contribute to improving colorectal cancer outcomes and mitigate disparities.

## Key Points

**Question:**

Is there variation in the diagnostic test completion rate and in the time to diagnostic workup among veterans younger than 50 years with iron-deficiency anemia (IDA) and/or hematochezia?

**Findings:**

In this cohort study of veterans with IDA and/or hematochezia, diagnostic testing rates were low. Diagnostic testing was less likely among female, Black, and Hispanic veterans with IDA and Hispanic veterans with hematochezia.

**Meaning:**

This study suggests that optimizing timely follow-up may help improve early age–onset colorectal cancer–related outcomes and reduce sex-based and race and ethnicity–based disparities.

## Teaser

This cohort study evaluates the rate of diagnostic endoscopy or colonoscopy and the time to testing among veterans younger than 50 years with iron-deficiency anemia (IDA) and hematochezia.

## Introduction

Colorectal cancer (CRC) is the second leading cause of cancer death in the United States.^1^ The proportion of CRC diagnosed among adults younger than 50 years of age—hereafter called early age–onset CRC (EAOCRC)—has increased over time, with cases among this group often diagnosed at later stages, requiring more intense treatment.^2,3,4,5,6,7,8,9,10^ About 70% to 95% of patients with EAOCRC present with concerning or “red-flag” signs or symptoms that could be indicative of CRC.^11,12^ Risk of EAOCRC is elevated up to 10-fold among individuals with a diagnosis of iron-deficiency anemia (IDA) or hematochezia.^13,14^ Given the frequency of symptomatic EAOCRC presentation, more aggressive workup of adults younger than 50 years of age with these conditions may enhance timely diagnosis and treatment and ultimately improve EAOCRC outcomes.^13,15^

Published guidelines recommend that all men and postmenopausal women with IDA undergo bidirectional endoscopy—both esophagogastroduodenoscopy and colonoscopy.^16,17^ The American Gastroenterological Association updated their guidelines in 2020 to additionally recommend that otherwise asymptomatic premenopausal women (eg, those without another explanation, such as menorrhagia) also receive bidirectional endoscopy.^18^ Adults younger than 50 years of age with hematochezia without lower abdominal symptoms and no known source of bleeding are also recommended to undergo colonoscopy.^19,20^ However, the extent to which these guidelines are followed, and the variation in following guidelines across populations, has not been widely studied, to our knowledge. Accordingly, our goal was to examine the receipt of guideline-concordant workup for IDA and hematochezia and the factors associated with any diagnostic workup for adults younger than 50 years with IDA and/or hematochezia.

## Methods

### Study Design, Setting, and Data Sources

We conducted a retrospective cohort study among US veterans aged 18 to 49 years receiving care between October 1, 1999, and December 31, 2019, in the Veterans Health Administration (VHA).^21^ To identify study data, we used several Department of Veterans Affairs (VA) electronic health record data resources, including the VA Corporate Data Warehouse, the VHA Vital Status file, and the National Death Index. We used separate IDA and hematochezia cohorts based on a prior study that examined these CRC symptoms separately.^14^ We excluded veterans with EAOCRC or inflammatory bowel disease diagnoses prior to diagnosis of IDA or hematochezia. Reporting on study design, analyses, and results followed the guidelines outlined by the Strengthening the Reporting of Observational Studies in Epidemiology (STROBE) reporting guideline and statement specific to cohort studies. The study was approved by the Veterans Affairs San Diego and University of California San Diego institutional review boards and was granted a waiver of informed consent by both institutional review board committees because the data are retrospective from a previously defined cohort with data collected under waivers of consent and Health Insurance Portability and Accountability Act authorization.

The IDA analytic cohort included veterans aged 18 to 49 years with a diagnosis of IDA, derived from a previously derived cohort of veterans who had at least 1 blood test measuring hemoglobin level conducted within the VHA.^14^ Iron-deficiency anemia was identified by laboratory diagnosis using World Health Organization criteria: a hemoglobin test identifying anemia (hemoglobin <13 g/dL for men and <12 g/dL for women [to convert to grams per liter, multiply by 10.0]) with a follow-up iron test within 3 months indicating iron deficiency (ferritin level ≤15 ng/mL [to convert to micrograms per liter, multiply by 1.0] or transferrin saturation level ≤16%).^18^ Veterans with menorrhagia or hysterectomy prior to or within 30 days of diagnosis of IDA were also excluded. In addition, we excluded veterans based on any *International Classification of Diseases, Ninth Revision* (*ICD-9*) and *International Statistical Classification of Diseases and Related Health Problems, Tenth Revision* (*ICD-10*) diagnosis codes for IDA prior to the date of hemoglobin blood test.

The hematochezia analytic cohort included veterans aged 18 to 49 years with a diagnosis of hematochezia, derived from a larger cohort of adults who had at least 1 *Current Procedural Terminology* code for an office visit initiating care within the VHA.^14^ Hematochezia was identified by *ICD-9* codes (569.3 and 578.1) or *ICD-10* codes (K62.5 and K92.1). We excluded veterans based on any diagnosis of hematochezia prior to the date of first VHA office visit.

We also developed a cohort of adults with both IDA and hematochezia as a secondary analysis to examine factors associated with diagnostic workup when individuals had both diagnoses. We defined joint exposure of IDA and hematochezia as diagnoses within 60 days of each other.

### Study Outcomes and Exposures

Our primary outcome was the time to diagnostic testing. For veterans with IDA, diagnostic test completion was defined as bidirectional endoscopy. To account for potential variation in practice where only the first endoscopic test performed would yield diagnostic resolution, we counted the first date of esophagogastroduodenoscopy or colonoscopy completion. For veterans with hematochezia, completed diagnostic testing was defined as either colonoscopy or sigmoidoscopy. In the secondary analysis of veterans with both IDA and hematochezia, diagnostic test completion was measured as completion of sigmoidoscopy or colonoscopy.

Candidate factors for timely diagnostic testing evaluated included age, measured continuously and categorically (18-29, 30-39, and 40-49 years), sex, patient-reported race and ethnicity (American Indian or Alaska Native, Asian or Pacific Islander, Hispanic, non-Hispanic Black, non-Hispanic White, other [multiracial, “other,” and unknown], and missing), US Census–defined region of VHA care (midwest, northeast, south, and west),^22^ and hemoglobin test value (measured in grams per deciliter) in the IDA cohort only. Inclusion of race and ethnicity as a variable in our study was to measure whether there were differences in time to diagnostic testing across race and ethnic groups.

### Statistical Analysis

Statistical analysis was conducted from August 2021 to August 2023. We conducted survival analyses to examine the association between candidate factors and time to diagnostic testing, including measurement of cumulative diagnostic test completion rate at 60 days, 180 days, and 2 years, based on a Kaplan-Meier estimation. Additional estimates of the cumulative diagnostic test completion rate were calculated by age group, sex, race and ethnicity, and census-defined geographic region. Follow-up was defined as the time from IDA and/or hematochezia diagnosis to 1 of the following: (1) endoscopy workup (outcome), (2) death, (3) end of 2 years of follow-up (730 days), or (4) December 31, 2019. The choice to end follow-up at a maximum of 2 years was based on the theory that diagnostic testing after this time would no longer correspond to the diagnosis of IDA and/or hematochezia. The proportional hazards assumption was tested by examining the correlation between time and scaled Schoenfeld residuals for all variables. Because the proportional hazards assumption was violated in all models, Poisson regression models were used to calculate rate ratios (RRs) and corresponding 95% CIs. Sensitivity analyses were performed excluding sigmoidoscopy as a recommended diagnostic examination for veterans with both IDA and hematochezia. All *P* values were from 2-sided tests and results were deemed statistically significant at *P* < .05. We used R, version 4.0.2 (R Project for Statistical Computing) to perform our analyses.^23^

## Results

Of 3 728 118 veterans aged 18 to 49 years with at least 1 encounter in the VHA health system between 1999 and 2019, 59 169 had an IDA diagnosis (mean [SD] age, 40.7 [7.1] years; 30 502 men [51.6%]), 189 185 had a hematochezia diagnosis (mean [SD] age, 39.4 [7.6] years; 163 690 men [86.5%]), and 2287 had both an IDA and hematochezia diagnosis (mean [SD] age, 41.6 [6.9] years; 1856 men [81.2%]) (Table 1). The overall population was predominantly male and non-Hispanic White and aged 40 to 49 years. Cumulative diagnostic test completion rates by symptom identified are shown in the Figure.

**Table 1.** Cohort Characteristics: Veterans Aged 18 to 49 Years With IDA or Hematochezia Between 1999 and 2019

Characteristic	Veterans, No. (%)		
	IDA cohort (n = 59 169)	Hematochezia cohort (n = 189 185)	IDA and hematochezia cohort (n = 2287)
Age, median (IQR), y	43.0 (36.0-47.0)	41.0 (33.0-46.0)	44.0 (38.0-47.0)
Age group, y			
<30	5910 (10.0)	27 682 (14.6)	191 (8.4)
30-39	15 540 (26.3)	54 773 (29.0)	495 (21.6)
40-49	37 719 (63.7)	106 730 (56.4)	1601 (70.0)
Sex			
Male	30 502 (51.6)	163 690 (86.5)	1856 (81.2)
Female	28 667 (48.4)	25 495 (13.5)	431 (18.8)
Race and ethnicity			
American Indian or Alaska Native	524 (0.9)	1436 (0.8)	31 (1.4)
Asian, Native Hawaiian, or Pacific Islander	963 (1.6)	3971 (2.1)	56 (2.5)
Hispanic	4161 (7.0)	17 137 (9.1)	202 (8.8)
Non-Hispanic Black	24 480 (41.4)	44 939 (23.8)	750 (32.8)
Non-Hispanic White	23 279 (39.3)	105 341 (55.7)	1030 (45.0)
Missing	4476 (7.6)	12 330 (6.5)	168 (7.4)
Other^a^	1286 (2.2)	4031 (2.1)	50 (2.2)
VHA region			
Midwest	9621 (16.3)	38 797 (20.5)	441 (19.3)
Northeast	6821 (11.5)	20 750 (11.0)	248 (10.8)
South	32 399 (54.8)	91 965 (48.6)	1168 (51.1)
West	10 328 (17.5)	37 673 (19.9)	430 (18.8)
Hemoglobin test value, median (IQR), g/dL	11.4 (10.4-11.9)	NA	11.1 (9.3-11.9)

Abbreviations: IDA, iron-deficiency anemia; NA, not applicable; VHA, Veterans Health Administration.

SI conversion factor: To convert hemoglobin to grams per liter, multiply by 10.0.

^a^ Includes individuals who are multiracial, categorized as “other” within the health record, or unknown.

**Figure.**
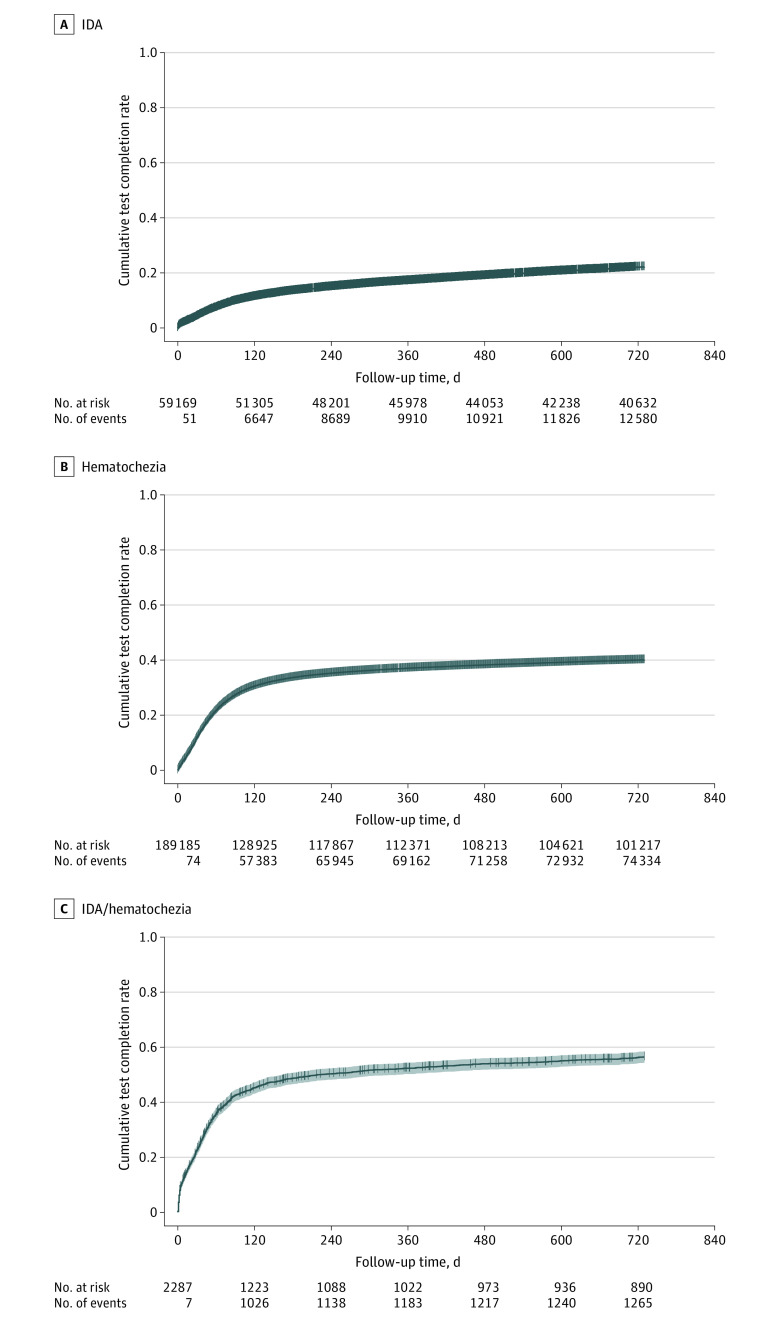
Kaplan-Meier Curves of Cumulative Diagnostic Test Completion Rates, by Diagnosed Symptom IDA indicates iron-deficiency anemia.

### Veterans With IDA

Of the 59 169 veterans with IDA, 37 719 (63.7%) were aged 40 to 49 years, 28 667 (48.4%) were women, 24 480 (41.4%) were classified as Black, and 4161 (7.0%) were classified as Hispanic (Table 1). The estimated cumulative diagnostic test completion rates were 7% (95% CI, 7%-8%) at 60 days and 22% (95% CI, 22%-22%) at the end of 2 years (Table 2), with a median 109 days (IQR, 40-311 days) to diagnostic testing among those completing a diagnostic test. Women had markedly lower cumulative diagnostic test completion rates at 60 days (women vs men: 3% [95% CI, 3%-3%] vs 11% [95% CI, 11%-12%]) and 2 years (13% [95% CI, 12%-13%] vs 31% [95% CI, 31%-32%]) (Table 2). Black veterans had lower cumulative diagnostic test completion rates compared with White veterans at 2 years (18% [95% CI, 17%-18%] vs 27% [95% CI, 16%-28%]). In Poisson models, adults aged 30 to 39 years (RR, 1.50; 95% CI, 1.37-1.64) and those aged 40 to 49 years (RR, 2.40; 95% CI, 2.22-2.61) were more likely to receive diagnostic testing compared with adults younger than 30 years (Table 3). Women had a lower likelihood of diagnostic testing (RR, 0.42; 95% CI, 0.40-0.43) compared with men. All non-White racial and ethnic groups had lower likelihoods of diagnostic testing compared with White veterans; non-Hispanic Black (RR, 0.65; 95% CI, 0.62-0.68) and Hispanic (RR, 0.88; 95% CI, 0.82-0.94) veterans had markedly lower likelihoods of diagnostic test completion. Adults with IDA receiving VHA care in the northeast (RR, 0.86; 95% CI, 0.80-0.91), south (RR, 0.80; 95% CI, 0.76-0.84), and west (RR, 0.92; 95% CI, 0.87-0.97) had lower likelihoods of diagnostic test completion compared with those in the midwest.

**Table 2.** Cumulative Endoscopy Completion Rates by Symptom at Time of Start of Follow-Up, Stratified by Age, Sex, and Race and Ethnicity

Characteristic	Cumulative endoscopy completion rate, % (95% CI)								
	IDA			Hematochezia			IDA and hematochezia		
	60 d	180 d	2 y	60 d	180 d	2 y	60 d	180 d	2 y
Overall	7 (7-8)	13 (13-14)	22 (22-22)	22 (22-22)	34 (33-34)	40 (40-40)	36 (34-38)	49 (47-51)	56 (54-58)
Age group, y									
<30	4 (4-5)	7 (6-8)	11 (10-11)	18 (18-19)	27 (27-28)	32 (31-32)	31 (24-37)	46 (38-53)	53 (45-60)
30-39	6 (5-6)	10 (10-11)	16 (16-17)	21 (21-21)	32 (31-32)	37 (37-37)	38 (34-42)	49 (45-54)	55 (51-60)
40-49	9 (8-9)	16 (15-16)	26 (26-27)	23 (23-23)	36 (36-36)	44 (43-44)	36 (34-38)	49 (46-52)	57 (55-60)
Sex									
Male	11 (11-12)	20 (19-20)	31 (31-32)	22 (22-22)	34 (33-34)	40 (40-40)	37 (35-39)	49 (47-51)	57 (55-59)
Female	3 (3-3)	7 (6-7)	13 (12-13)	22 (21-22)	34 (33-34)	40 (39-40)	33 (28-37)	48 (43-52)	53 (48-58)
Race and ethnicity									
American Indian or Alaska Native	7 (5-9)	11 (9-14)	22 (18-25)	21 (19-23)	32 (30-34)	39 (37-42)	32 (14-47)	52 (31-67)	56 (34-70)
Asian, Native Hawaiian, or Pacific Islander	9 (7-11)	15 (13-17)	22 (19-24)	23 (21-24)	34 (33-36)	40 (38-41)	30 (17-42)	43 (29-55)	47 (32-59)
Hispanic	9 (8-10)	15 (14-16)	24 (23-25)	20 (20-21)	33 (32-33)	39 (38-40)	41 (34-37)	53 (46-60)	61 (54-67)
Non-Hispanic Black	5 (5-5)	10 (10-10)	18 (17-18)	20 (20-21)	33 (32-33)	40 (40-40)	33 (29-36)	45 (41-48)	54 (50-57)
Non-Hispanic White	10 (9-10)	17 (17-18)	27 (26-28)	23 (23-23)	35 (34-35)	41 (40-41)	38 (35-41)	51 (48-54)	58 (55-61)
Missing	7 (6-8)	12 (11-13)	21 (19-22)	20 (19-20)	30 (30-31)	36 (35-37)	37 (29-44)	47 (39-54)	54 (46-62)
Other^a^	7 (6-9)	13 (12-15)	23 (20-25)	21 (20-22)	33 (31-34)	39 (38-41)	46 (30-58)	58 (42-70)	60 (44-72)
VHA region									
Midwest	9 (9-10)	16 (15-17)	26 (25-27)	22 (22-23)	33 (32-33)	39 (39-39)	38 (33-42)	50 (47-53)	56 (51-60)
Northeast	8 (7-8)	13 (12-14)	22 (21-23)	23 (22-24)	34 (33-34)	40 (39-41)	33 (27-39)	43 (37-49)	54 (47-60)
South	6 (6-7)	12 (12-13)	21 (20-21)	22 (22-22)	35 (34-35)	42 (41-42)	35 (32-38)	50 (47-53)	58 (55-61)
West	8 (8-9)	15 (14-16)	24 (23-25)	20 (20-21)	32 (31-32)	38 (37-38)	38 (33-43)	50 (45-54)	55 (49-59)

Abbreviations: IDA, iron-deficiency anemia; VHA, Veterans Health Administration.

^a^ Includes individuals who are multiracial, categorized as “other” within the health record, or unknown.

**Table 3.** Likelihood of Colonoscopy Completion, Stratified by Symptom at Time of Start of Follow-Up

Characteristic	Rate ratio (95% CI)		
	IDA	Hematochezia	IDA and hematochezia
Age, y			
<30	1 [Reference]	1 [Reference]	1 [Reference]
30-39	1.50 (1.37-1.64)	1.15 (1.12-1.18)	1.04 (0.83-1.32)
40-49	2.40 (2.22-2.61)	1.36 (1.33-1.40)	1.07 (0.88-1.33)
Sex			
Male	1 [Reference]	1 [Reference]	1 [Reference]
Female	0.42 (0.40-0.43)	0.99 (0.97-1.01)	0.94 (0.82-1.09)
Race and ethnicity			
American Indian or Alaska Native	0.80 (0.66-0.96)	0.95 (0.88-1.04)	0.96 (0.57-1.50)
Asian, Native Hawaiian, or Pacific Islander	0.80 (0.69-0.92)	0.97 (0.92-1.02)	0.81 (0.53-1.18)
Hispanic	0.88 (0.82-0.94)	0.96 (0.93-0.98)	1.05 (0.86-1.27)
Non-Hispanic Black	0.65 (0.62-0.68)	0.99 (0.97-1.00)	0.93 (0.82-1.05)
Non-Hispanic White	1 [Reference]	1 [Reference]	1 [Reference]
Missing	0.72 (0.67-0.78)	0.88 (0.85-0.91)	0.91 (0.72-1.13)
Other^a^	0.84 (0.74-0.94)	0.96 (0.92-1.01)	1.05 (0.71-1.49)
VHA region			
Midwest	1 [Reference]	1 [Reference]	1 [Reference]
Northeast	0.86 (0.80-0.91)	1.02 (1.00-1.05)	0.97 (0.78-1.20)
South	0.80 (0.76-0.84)	1.06 (1.04-1.08)	1.03 (0.89-1.20)
West	0.92 (0.87-0.97)	0.96 (0.94-0.98)	0.98 (0.82-1.17)
Hemoglobin test value (g/dL)	0.95 (0.93-0.96)	NA	1.02 (0.99-1.04)

Abbreviations: IDA, iron-deficiency anemia; NA, not applicable; VHA, Veterans Health Administration.

^a^ Includes individuals who are multiracial, categorized as “other” within the health record, or unknown.

### Veterans With Hematochezia

Among the 189 185 veterans with hematochezia, most were aged 40 to 49 years (106 730 [56.4%]) and men (163 690 [86.5%]) and were composed of 105 341 White (55.7%), 44 939 Black (23.8%), and 17 137 Hispanic (9.1%) veterans (Table 1). The estimated cumulative diagnostic test completion rates were 22% (95% CI, 22%-22%) at 60 days and 40% (95% CI, 40%-40%) at the end of 2 years (Table 2), with the median time to diagnostic testing being 53 days (IQR, 26-111 days) among those completing workup. Adults aged 30 to 39 years (RR, 1.15; 95% CI, 1.12-1.18) and adults aged 40 to 49 years (RR, 1.36; 95% CI, 1.33-1.40) had a greater likelihood of diagnostic test completion compared with adults younger than 30 years (Table 3). Hispanic veterans (RR, 0.96; 95% CI, 0.93-0.98) with hematochezia had a lower likelihood of diagnostic test completion compared with White veterans. Adults with hematochezia in the west (RR, 0.96; 95% CI, 0.94-0.98) had a lower likelihood of diagnostic test completion, whereas those in the south (RR, 1.06; 95% CI, 1.04-1.08) had a higher likelihood of diagnostic test completion compared with those in the midwest.

### Veterans With IDA and Hematochezia

There were 2287 adults with a diagnosis of both IDA and hematochezia (Table 1). Veterans with both IDA and hematochezia were mostly aged 40 to 49 years (1601 [70.0%]) and men (1856 [81.2%]), with a large proportion of Black veterans (750 [32.8%]). The estimated cumulative diagnostic test completion rates were 36% (95% CI, 34%-38%) at 60 days and 56% (95% CI, 54%-58%) at the end of 2 years, with a median follow-up of 41 days (IQR, 12-85 days) among those completing diagnostic testing (Table 2). Removing sigmoidoscopy as a diagnostic examination was associated with slightly lower 60-day and 2-year cumulative diagnostic test completion rates (eTable in Supplement 1).

## Discussion

Among veterans aged 18 to 49 years with a diagnosis of IDA and/or hematochezia, the cumulative proportion receiving guideline-recommended diagnostic testing after 2 years was low, at 22% for those with IDA and 40% for those with hematochezia. Furthermore, receipt of diagnostic testing within 60 days of diagnosis of IDA or hematochezia occurred for only 7% of those with IDA and 22% of those with hematochezia. After IDA diagnosis, men were more likely to receive diagnostic testing compared with women, and the likelihood of workup increased with age. After diagnosis of hematochezia, adults aged 30 to 49 years were more likely to receive diagnostic testing compared with adults younger than 30 years. Black veterans were less likely to receive diagnostic testing after an IDA diagnosis, and Hispanic veterans were less likely to receive diagnostic testing after an IDA or hematochezia diagnosis, compared with White veterans. Given that both IDA and hematochezia have been shown to increase EAOCRC risk, our findings suggest that there are significant opportunities to improve EAOCRC outcomes, including sex-based and race and ethnicity–based disparities, by promoting diagnostic testing after IDA and hematochezia diagnosis.

Men were more likely than women to receive diagnostic testing after an IDA diagnosis, which aligns with prior research.^24^ Although the risk of incident and fatal EAOCRC is lower among women than men,^25,26^ the markedly lower likelihood of diagnostic testing is surprising, given that the population with a diagnosis of IDA had a nearly equal amount of men and women. Iron-deficiency anemia is commonly attributed to menorrhagia in premenopausal women, but our study found the disparity in workup persisted even after removal of women with diagnosed cases of menorrhagia or prior hysterectomy. It is plausible that clinicians are more likely to attribute iron deficiency in women to disorders of menstruation. Nevertheless, more research is necessary to uncover why this sex-based disparity in diagnostic follow-up exists and whether substantially lower rates of endoscopic follow-up for women are clinically appropriate.

Our study found that Black veterans with IDA were less likely to receive diagnostic testing than White veterans. Black veterans of all ages have been shown to have higher CRC incidence and mortality and a more advanced stage of CRC at presentation.^2^ Recent evidence indicates that, despite greater increases in EAOCRC incidence among White adults compared with Black adults over a 15-year period, White adults had higher relative survival.^27^ Differences between Black and White adults have been postulated to be associated with differences in risk factor burden, access to health care, and follow-up patterns, such as for abnormal results from stool tests performed for CRC screening, as well as structural racism. Our findings of variation in diagnostic testing after IDA and hematochezia diagnosis suggest that there may be specific clinical scenarios amenable for interventions that can reduce disparities in CRC outcomes for Black vs White adults.

Hispanic veterans with IDA and/or hematochezia were also found to have a lower likelihood of receiving diagnostic testing compared with White veterans. Recent evidence has shown that EAOCRC incidence is increasing rapidly among Hispanic adults, particularly for regional or distant-stage disease.^26,28,29^ In addition, Hispanic adults have similar or worse EAOCRC-related survival compared with White adults.^29,30,31^ Our study findings indicate that effective strategies for timely diagnostic testing may help address these differences.

To date, there are few data on race and ethnicity–specific diagnostic follow-up patterns for CRC-related conditions, particularly for adults with IDA and/or hematochezia, to our knowledge. Prior studies that examined colonoscopy follow-up after positive stool blood test results found that Black veterans were more likely to receive follow-up than White veterans, including 1 study among adults aged 45 to 50 years.^32,33^ Similarly, Hispanic veterans were found to have higher rates of diagnostic colonoscopy completion after abnormal stool blood test results compared with non-Hispanic adults.^34^ These findings in diagnostic follow-up after abnormal stool blood test results contradict the diagnostic testing findings in our study, in which Black veterans and Hispanic veterans had a lower likelihood of receiving diagnostic testing. Other studies in community health settings have found no significant association between race and ethnicity and diagnostic colonoscopy workup for adults with positive stool blood test results.^35^ Although these studies highlight the variation in findings regarding race and ethnicity–specific follow-up patterns, there is still limited evidence focusing on follow-up after potential signs or symptoms for CRC.^29^

Our study was conducted within the VHA, where all participants had no barriers associated with insurance coverage, yet disparities still were found. The observed disparities may be associated with individual (social factors such as transportation), interpersonal (such as family circumstances and interpersonal bias in health care), organizational, community, or societal influences.^36,37^ It is also plausible that racial and ethnic differences could be due to variations in adherence to clinician follow-up recommendations potentially in response to experiences of structural racism in health care. Explicit and implicit bias by health care professionals and health care systems in the assessment of and management recommendations for Black or Hispanic adults vs White adults with IDA and/or hematochezia have been documented in the literature.^38,39,40^ Nevertheless, our results suggest that more research is necessary to understand whether race and ethnicity may be associated with the response of clinicians or the health care system to these diagnoses. Reasons for differences in diagnostic follow-up for IDA and hematochezia, as well as interventions to address these differences, require further study and could help to address racial and ethnic disparities in CRC outcomes.

### Limitations and Strengths

Our study has some limitations. Given the limitations in capturing community health data, we were unable to ascertain endoscopy uptake claims outside of the VHA, which could underestimate diagnostic testing completion rates. Nonetheless, we do not expect these ascertainment issues to explain the between-group variation in testing rates. In addition, our study was unable to examine specific individual-, clinician-, or system-related factors, such as distance to care and interpersonal bias, that may contribute to the observed findings. Future research should focus on whether these factors are associated with disparities in diagnostic test completion.

Our study also has some strengths. One key strength was that the absolute numbers of women in our IDA (n = 28 667) and hematochezia (n = 25 495) cohorts were substantial, and, to our knowledge, they represent one of the largest observations of diagnostic testing outcomes among women younger than 50 years presenting with IDA or hematochezia.

## Conclusions

This cohort study found that the rates of diagnostic testing with endoscopy among veterans aged 18 to 49 years with IDA and/or hematochezia are low and vary significantly across demographic groups. Black veterans with IDA and Hispanic veterans with IDA and/or hematochezia were less likely than White veterans to receive diagnostic testing for EAOCRC. Furthermore, women with IDA were less likely than men to receive diagnostic testing. Optimizing diagnostic test completion among individuals with IDA and/or hematochezia may help improve early detection of EAOCRC and contribute to reducing EAOCRC-related disparities.

## Notes

Supplement 1. **eTable.** Sensitivity Analysis of Characteristics of Veterans Age 18-49 With vs Without Timely Diagnostic Workup After Concurrent Diagnosis With Both IDA and Hematochezia, Excluding Sigmoidoscopy (N=2,276)

Supplement 2.
Data Sharing Statement
