# Supplementary material for: Time to Endoscopy or Colonoscopy Among Adults Younger Than 50 Years With Iron-Deficiency Anemia and/or Hematochezia in the VHA
Source: JAMA Netw Open. 2023 Nov 6;6(11):e2341516. doi: 10.1001/jamanetworkopen.2023.41516 (PMC10628727; doi:10.1001/jamanetworkopen.2023.41516)
Supplement: Supplement 1. — eTable. Sensitivity Analysis of Characteristics of Veterans Age 18-49 With vs Without Timely Diagnostic Workup After Concurrent Diagnosis With Both IDA and Hematochezia, Excluding Sigmoidoscopy (N=2,276) [file jamanetwopen-e2341516-s001.pdf]

Supplementary Online Content

Demb J, Liu L, Murphy CC, Doubeni CA, Martinez ME, Gupta S. Time to endoscopy or colonoscopy among adults younger than 50 years with iron-deficiency anemia and/or hematochezia in the VHA. *JAMA Netw Open.* 2023;6(11):e2341516. doi:10.1001/jamanetworkopen.2023.41516

eTable. Sensitivity Analysis of Characteristics of Veterans Age 18-49 With vs Without Timely Diagnostic Workup After Concurrent Diagnosis With Both IDA and Hematochezia, Excluding Sigmoidoscopy (N=2,276)

This supplementary material has been provided by the authors to give readers additional information about their work.

© 2023 Demb J et al. *JAMA Network Open.*

eTable. Sensitivity Analysis of Characteristics of Veterans Age 18-49 With vs Without Timely Diagnostic Workup After Concurrent Diagnosis With Both IDA and Hematochezia, Excluding Sigmoidoscopy (N=2,276)

	IDA and Hematochezia		
	**60 days % (95% CI)**	**180 days % (95% CI)**	**2 years % (95% CI)**
**Overall**	34 (32-36)	46 (44-48)	53 (51-55)
**Age Group**			
**<30**	28 (22-35)	42 (34-48)	48 (40-54)
**30-39**	36 (31-40)	45 (41-50)	51 (46-55)
**40-49**	34 (32-36)	47 (44-49)	55 (52-57)
**Sex**			
**Male**	35 (32-37)	46 (44-49)	54 (52-57)
**Female**	30 (26-34)	44 (39-48)	49 (44-54)
**Race/Ethnicity**			
**American Indian or Alaska Native**	29 (11-43)	42 (22-58)	50 (28-65)
**Asian, Native Hawaiian, or Pacific Islander**	30 (14-42)	41 (27-53)	45 (30-57)
**Hispanic**	39 (32-45)	51 (43-57)	58 (51-65)
**Non-Hispanic Black**	31 (27-34)	43 (39-46)	51 (47-54)
**Non-Hispanic White**	36 (33-38)	48 (45-51)	55 (52-58)
**Missing**	33 (25-39)	42 (34-49)	51 (43-58)
**Other[a]**	46 (30-58)	52 (36-64)	54 (38-66)
**VHA Region**			
**Midwest**	36 (32-41)	47 (42-51)	54 (49-58)
**Northeast**	34 (27-39)	41 (35-47)	50 (44-56)
**South**	33 (30-35)	47 (44-49)	54 (51-57)
**West**	36 (31-40)	46 (42-51)	52 (47-57)

Abbreviations: IDA, iron-deficiency anemia; VHA, Veterans Health Administration

[a]Includes individuals who are multiracial, categorized as “other” within the health record, or unknown

© 2023 Demb J et al. *JAMA Network Open.*
