# Supplementary material for: Time to Endoscopy or Colonoscopy Among Adults Younger Than 50 Years With Iron-Deficiency Anemia and/or Hematochezia in the VHA
Source: JAMA Netw Open. 2023 Nov 6;6(11):e2341516. doi: 10.1001/jamanetworkopen.2023.41516 (PMC10628727; doi:10.1001/jamanetworkopen.2023.41516)
Supplement: Supplement 2. — Data Sharing Statement [file jamanetwopen-e2341516-s002.pdf]

Data Sharing Statement

Demb. Time to Endoscopy or Colonoscopy Among Adults Younger Than 50 Years With Iron-Deficiency Anemia and/or Hematochezia in the VHA. *JAMA Netw Open*. Published November 06, 2023. doi:10.1001/jamanetworkopen.2023.41516

Data
Data available: No

Additional Information
Explanation for why data not available: The data used for this study are not publicly available. Access to VHA data requires appointment to work within the VHA system and further approval of study protocol and data access requests. The study protocol and programming code for analyses are available upon request.
